# The Ghosts of COVID-19: Lost between the Waves

**DOI:** 10.4269/ajtmh.22-0352

**Published:** 2022-11-14

**Authors:** Sitarah Mathias

**Affiliations:** Boston Children’s Hospital, Boston, Massachusetts

I remember the evening Mr. X was wheeled into our emergency room (ER) in a little nonprofit missionary hospital in rural South India. He presented with acute-onset neurological deficits that were rapidly deteriorating and his blood pressure was soaring. The monsoons had just begun, the COVID-19 lockdowns had finally lifted, and our ER, staffed by one physician (me) and one nurse, was brimming with sick patients despite the torrential rainfall battering the little village we were in. While I attended to Mr. X, his sobbing relatives gave me his history. It took me less than a minute to realize that Mr. X was yet another ghost victim of COVID-19.

The summer 2020 lockdown disrupted supply chains, including those for essential medication and hospital equipment, and Mr. X was one of those unfortunate patients who had lost his antihypertensive medication supply. Feeling no different after a few days sans medication, he believed he had been “cured.” Therefore, when supply chains were reestablished, his treatment was not. Now, a month later, he lay in the ER while his family tried in vain to arrange for his impending referral to a tertiary center.

The village where I worked was flanked by a major south Indian metropolis and two smaller cities, yet was poorly connected by rail and road. Although it had its fair share of government and private medical facilities to access basic medical, pediatric, emergency, orthopedic, general surgery, and obstetric care, critical emergent treatment and subspecialty services were only available at tertiary care hospitals in these surrounding cities—often an hour or more away by road. Government ambulances were available to ferry patients to higher centers for free; however, their overworked status and predetermined referral routes meant that private ambulances were often called for more tailored service. These private enterprises came with a hefty price tag, regularly costing patient families between 30% and 50% of their monthly household income for one referral. In addition, COVID-19 policies and other costs during the height of the pandemic meant that finding a free or affordable intensive care unit bed in a tertiary care hospital was almost impossible, particularly because most health expenditures here are out-of-pocket. I watched Mr. X’s family struggle to negotiate prices with potential ambulance drivers and hospitals, forced to weigh the life of the family patriarch against their possessions and options. Watching these events unfold made it clear to me that society—through policies and actions—had not only failed him these past months, but also continued to do so when he was most vulnerable.

As tragic as it is when society and policies fail my patients, a lack of basic medical resources to treat my patients is tremendously more devastating. That is how I met Mrs. A. She presented to the ER one night with acute pulmonary edema complicating her congestive heart failure. With only two other staff nurses in the whole hospital, I strongly advised a referral to a tertiary care center, but her family stoutly refused to move her before dawn for logistic reasons. After admitting her to the high-dependency unit at our hospital, I realized that our supply of furosemide had run out. The last emergency vial had been used earlier that day, and the fresh stock delivery had been delayed—more fallout from the COVID-19–related supply chain disruptions. The three of us scoured every floor till we found two vials—the last two—in the delivery ward. Our short-lived relief was replaced by yet another obstacle: our only working infusion pump had stopped working and all repair services had been delayed because of the pandemic.

Determined to keep her alive until she could be referred, I hunted down a pediatric intravenous drip set and titrated the appropriate infusion rate by manually counting and timing furosemide drops. All night I ran between the second-floor high-dependency unit and the first-floor ER, splitting my time between Mrs. A and other patients. Against all odds, she improved overnight. As dawn broke, the new light brought with it her family and funds, and she was referred to the tertiary hospital, alive. I watched the retreating ambulance as the rising sun draped the trees and fields in its golden rays that morning, realizing how close we had come to failing yet another patient because of the ripple effects of the pandemic.

I do not know if either of these patients survived their ordeals. What I do know is that society failed them at individual and collective levels. Mr. X and Mrs. A both represent a uniquely vulnerable population—not impoverished enough to reap the benefits of multiple government schemes for the poor, yet not privileged enough to be able to access health care on their own terms. For years, as a society, we have strived to create opportunities to serve these underserved populations at local and global levels. We have organized outreach and health camps, set up health centers in remote and dangerous locations—all to care for those who desperately need it. Yet during the pandemic, we set in motion a cascade of events that neglected both these vulnerable populations and our existing responsibilities. Mr. X and Mrs. A were only two of the many patients with preventable strokes, myocardial infarctions, decompensated congestive heart failure, chronic obstructive pulmonary disease, renal failure, and hyperglycemic complications that I treated during the pandemic. In addition to scant supplies, elective hospital and outpatient services were also limited during the 2020 lockdown period to reduce the spread of COVID-19. This, together with the public fear of contracting COVID-19 from hospital settings, led to the loss of preventable health-care monitoring and treatment of many individuals with chronic diseases. The hoarding of available medical stock to inflate prices and profit margins artificially only compounded the situation. The casualties of these unfortunate events were not apparent until after the lockdown, when they emerged in the form of decompensated patients—patients with uncontrolled disease or patients who had developed avoidable, irreversible complications. Almost every one of them had lost access to medication and follow-up care from either the disruption in health services and/or the loss of household income. Although this cohort did not ultimately succumb to COVID-19 during the pandemic waves, they are still victims of the pandemic fallout, forgotten by the masses, unaccounted for by statisticians, and ignored by politicians. They are the ghosts of COVID-19, lost between the waves. In our single-issue view of the pandemic, we as a community neglected our routine responsibilities, leaving a trail of tragedy from its fallout in the form of lack of access to health care. And for once, this lack of accessibility could not be attributed solely to geographic and/or financial barriers.

Although this inter-COVID-19–wave tragedy is not unique to low- and middle-income countries, it is important to recognize these uncounted COVID-19 “ghost” casualties. These “lost statistics” suffered needlessly and paid the price for our restricted response to a stressful global event. The best way to honor them is not only to acknowledge this, but also to be more deliberate in adopting holistic approaches that do not allow anyone to slip through the cracks.

